# Rapid On-Site Detection of Clarithromycin in Water Using an Evanescent Wave Fluorescence Immunosensor

**DOI:** 10.3390/bios16100588

**Published:** 2026-10-20

**Authors:** Wenjing Zhang, Yuru Liu, Jingjing Liu, Feng Long, Hejie Li, Xinyu Zhang, Yuxiang Shi, Chunsheng Li

**Affiliations:** 1 Cell Biochemistry Laboratory, Biology Institute of Hebei Academy of Sciences, Shijiazhuang 050081, China; 17325262774@163.com (W.Z.); 2024111067@stu.hebust.edu.cn (Y.L.); sxndliujing@126.com (J.L.); n15227019149@163.com (H.L.); 2 College of Life Science and Food Engineering, Hebei University of Engineering, Handan 056038, China; zxinyu0115@163.com; 3 School of Food Science and Biology, Hebei University of Science and Technology, Shijiazhuang 050018, China; 4 School of Chemistry and Life Resources, Renmin University of China, Beijing 100872, China; longf04@ruc.edu.cn

**Keywords:** evanescent wave fluorescence, clarithromycin, immunosensor, water environment, on-site detection

## Abstract

Clarithromycin (CLA), a widely used macrolide antibiotic and contaminant of emerging concern (CEC), is frequently detected in aquatic environments. Its residues can induce bacterial antibiotic resistance, threatening public health and ecosystems. In this study, we constructed an evanescent wave fluorescence immunoassay for the rapid detection of clarithromycin. It detects CLA in water quickly, sensitively, and in a portable format. The sensor combines evanescent wave fluorescence with an indirect competitive immunoassay. It uses a CLA–bovine serum albumin (BSA) conjugate on an optical fiber bioprobe, along with a Cy5.5-labeled anti-clarithromycin monoclonal antibody (*K*_a_ = 7.40 × 10^9^ L/mol) as the signal reporter. After optimization, the assay gave a linear range of 7.50–77.45 μg/L. The LOD was 1.67 μg/L, and the LOQ was 7.50 μg/L. The functionalized fiber bioprobe also held up well in repeated use. It stayed stable for over 200 regeneration cycles, with an RSD of 5.63%. The entire automated detection workflow was completed within 20 min without manual intervention. Real-sample validation was performed using tap water and river water spiked at 10, 30, and 60 μg/L, yielding recoveries ranging from 87.13% to 104.84%, with relative standard deviations below 13.08%. These results were corroborated by ELISA. These attributes, combined with the instrument’s portability and the probe’s regenerability, render this biosensor a promising analytical platform for on-site environmental monitoring of clarithromycin residues.

## 1. Introduction

Clarithromycin (CLA), a 14-membered macrolide antibiotic, is widely prescribed for the treatment of respiratory tract infections, skin and soft tissue infections, and chronic pulmonary diseases [1,2,3,4,5]. Its bacteriostatic mechanism involves the suppression of bacterial protein synthesis by binding to the 50S ribosomal subunit [6,7,8], but improper use and residues of CLA come with real risks. For one thing, the misuse of clarithromycin can easily lead to resistant bacterial strains. These strains carry resistance genes that can move between organisms via horizontal gene transfer, which reduces the drug’s therapeutic potential [8,9,10,11]. For another, clarithromycin that is not fully absorbed by the human body or animals will enter the environment in the form of the parent drug or its metabolites through excreta [8,12]. It will not only contaminate water bodies such as surface water and domestic water but also directly affect the diversity and activity of soil microorganisms, thereby posing a potential threat to public health [13]. Long-term exposure of humans and animals to environments containing clarithromycin can raise the risk of illness and morbidity, and if this is not controlled, it will keep posing a serious and long-lasting threat to human health [9,10,14].

Liquid chromatography–tandem mass spectrometry (LC–MS/MS) is currently regarded as the gold standard for macrolide antibiotic quantification, offering exceptional sensitivity, selectivity, and reliability [11,15,16]. Nevertheless, LC–MS/MS-based protocols are constrained by complex sample pretreatment requirements, high operational costs, bulky instrumentation, and the necessity for skilled personnel, rendering them poorly suited for rapid on-site screening. Enzyme-linked immunosorbent assay (ELISA) represents an alternative immunochemical approach for CLA detection [17]. However, conventional ELISA is time-consuming, requires plate readers that lack portability, and is susceptible to matrix-induced false positives, which similarly limit its deployment in field settings. Given these concerns, the development of reliable analytical methods for CLA monitoring in environmental water is imperative.

Biosensor technologies have emerged as powerful analytical tools that bridge the gap between laboratory precision and field applicability, offering favorable specificity, high sensitivity, and point-of-care testing capability [18]. Fluorescent aptasensors based on fluorescent dyes and nanomaterials are widely studied for antibiotic detection. Aptamers show good stability and are easy to synthesize chemically. However, practical commercialization remains challenging, as well-validated high-affinity aptamers remain scarce, background and autofluorescence create interference, and their performance in complex real matrices is often unsatisfactory [19,20,21]. Among optical biosensing platforms, evanescent wave (EW) biosensors exploit the total internal reflection phenomenon at the core–cladding interface of an optical fiber to generate an exponentially decaying evanescent field into the surrounding medium. These devices integrate specific bioaffinity interactions with fluorescence-labeled antibody methods and utilize the favorable linear relationship between fluorescence signals and the concentration of the target analyte. That is how they achieve the accurate quantitative detection of target substances [22]. Accordingly, evanescent wave biosensors exhibit considerable application potential in the fields of environmental monitoring, medical diagnosis, and other related areas [23]. Evanescent wave biosensors present multiple attractive characteristics, including rapid analysis, high sensitivity, convenient portability, and low cost [24,25]. This technology has already been used to detect heavy metal ions [26,27], foodborne pathogenic microorganisms [28,29], and pesticide residues [30]. Nevertheless, evanescent-wave fluorescent immunosensors reported to date have been directed largely at veterinary drugs or common antibiotics. Wang et al., for example, built a field-deployable automated evanescent-wave fluorescence optofluidic biochip for the ultrasensitive determination of clenbuterol in complex animal urine matrices [22]. Zhuo et al. used an evanescent-wave biosensor to rapidly detect norfloxacin in water [31]. Quantitative determination of clarithromycin in environmental water samples, however, has not yet been reported.

**Figure 1:**
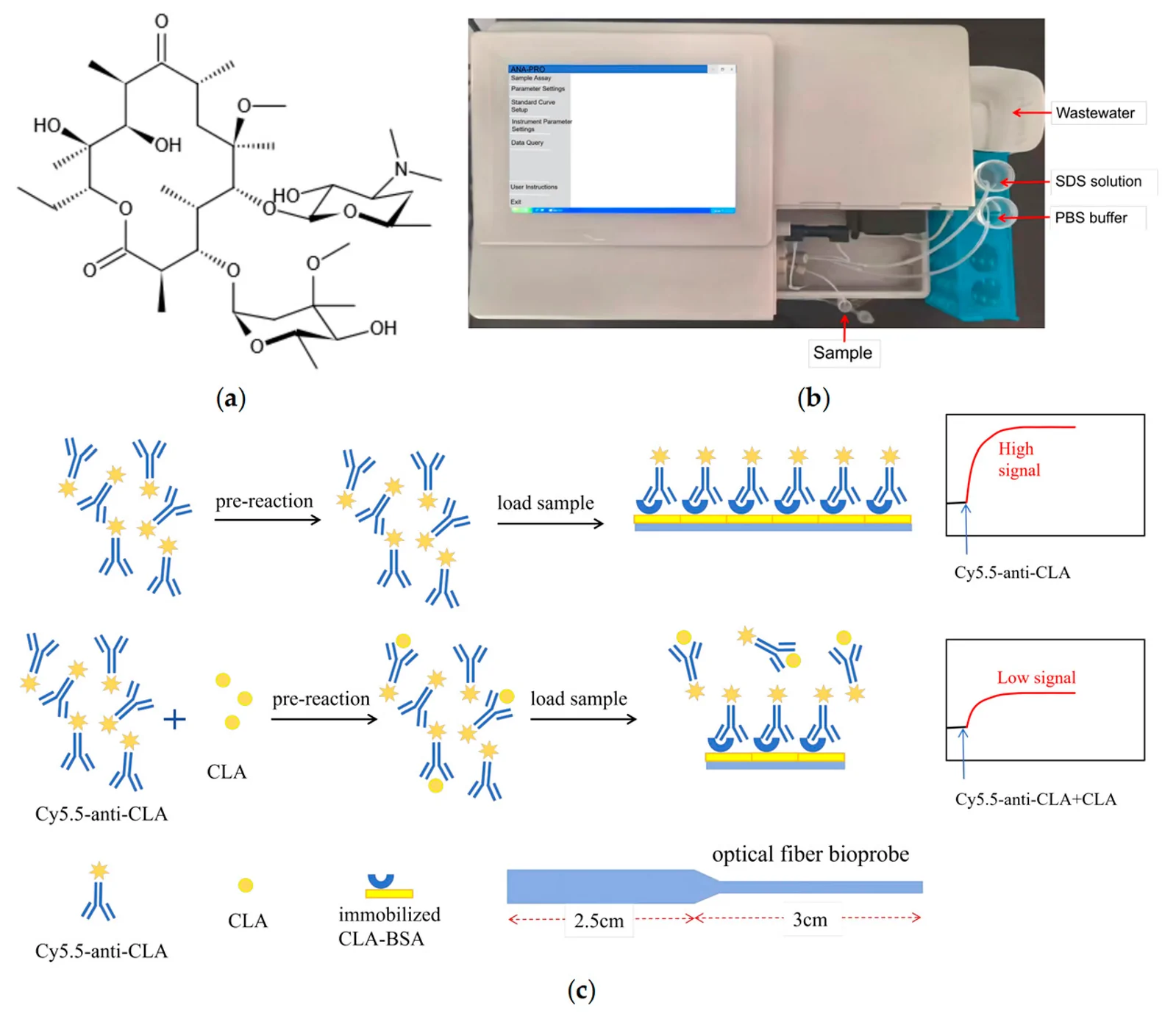
(**a**) The chemical structure of clarithromycin, (**b**) the evanescent wave biosensor, and (**c**) the indirect competitive immunoassay principle for CLA detection using the evanescent wave biosensor.

In this work, we describe the construction and analytical validation of an evanescent wave fluorescence immunosensor (Figure 1b) dedicated to the rapid on-site quantification of CLA in water samples. The sensor architecture comprises a CLA–BSA conjugate-immobilized optical fiber bioprobe and a Cy5.5-labeled anti-clarithromycin monoclonal antibody. By harnessing the indirect competitive immunoassay principle (Figure 1c), the biosensor translates CLA concentration into an inversely correlated fluorescence signal. We systematically characterized the antibody affinity and specificity. We also optimized the assay parameters, established the standard curve, and evaluated the regenerability of the probe. We validated the method in real water matrices. The resulting platform achieves a low detection limit and completes an assay in under 20 min. Its probe remains durable for more than 200 cycles. These features position the platform as a practical solution for on-site environmental surveillance of macrolide antibiotics.

## 2. Materials and Methods

### 2.1. Reagents and Materials

Clarithromycin (CLA) standard was purchased from Weiyel Inc. (Beijing, China). HRP-labeled goat anti-mouse IgG was purchased from Zhongshan Golden Bridge Biotechnology Co., Ltd. (Beijing, China). Hydrofluoric acid (30%, *v*/*v*) was obtained from Tianjin Hengxing Chemical Reagent Co., Ltd. (Tianjin, China). Hydrogen peroxide (30%, *v*/*v*) was purchased from Tianjin Zhengcheng Chemical Co., Ltd. (Tianjin, China). Toluene was obtained from Tianjin FengChuan Chemical Reagent Co., Ltd. (Tianjin, China). 4-Maleimidobutyric acid N-hydroxysuccinimide ester (GMBS), bovine serum albumin (BSA), and (3-mercaptopropyl) trimethoxysilane (MTS) were purchased from Sigma–Aldrich (St. Louis, MO, USA). Sodium dodecyl sulfate (SDS) was sourced from MP Biomedicals, LLC (Santa Ana, CA, USA). Cy5.5-N-hydroxysuccinimide ester (Cy5.5-SE) was obtained from MedChemExpress (Monmouth Junction, NJ, USA). Clarithromycin antigen (CLA-BSA) and antibody (CLA-4D3) were prepared in-house in our laboratory. Phosphate-buffered saline (PBS, 0.01 mol/L, pH = 7.4), PBST (PBS supplemented with 0.05% *v*/*v* Tween-20), 0.5% SDS regeneration solution (pH = 1.9), carbonate–bicarbonate buffer (CBS, pH = 9.3), and antibody dilution buffer were prepared in-house. A CLA stock solution (3.0 mg/mL in N,N-dimethylformamide) was prepared gravimetrically and stored at −20 °C. All other chemicals were of analytical grade or higher. All experiments used ultrapure water (resistivity ≥ 18.2 MΩ·cm).

### 2.2. Instrumentation

The evanescent wave biosensor platform was built in-house. It consisted of a 635 nm diode laser as the excitation source, a multiport rotary valve, a peristaltic pump, a photomultiplier tube (PMT) detector, and a mini-computer that handled fluidic control and data acquisition. Excitation light entered a multimode silica optical fiber, and it traveled through the fiber by total internal reflection. The fiber collected the fluorescence emitted by surface-bound Cy5.5, which was then spectrally filtered and converted into an electrical signal by the PMT. The instrument ran under automated sequence control, and its assay steps—baseline stabilization, sample incubation, regeneration, and rinsing—proceeded without manual intervention.

We used a spectrophotometer to measure UV–visible absorbance for antibody labeling characterization. We used a microplate reader for ELISA validation.

### 2.3. Preparation of the Functionalized Fiber Bioprobe

The optical fiber bioprobe was made following a previously reported GMBS crosslinking protocol [30] with a few small changes. First, we took a 5.5 cm piece of multimode optical fiber (600 μm core diameter) and mechanically stripped it to expose a 3.0 cm sensing region. The exposed region was etched in 30% (*v*/*v*) hydrofluoric acid to reduce the diameter to approximately 225 μm. The etched fiber was treated with piranha solution (concentrated H_2_SO_4_: 30% H_2_O_2_ = 3:1, *v*/*v*) for 30 min at room temperature, rinsed with ultrapure water and sonicated to reach a neutral state. After drying, the fiber was silanized in 2% (*v*/*v*) MTS in anhydrous toluene at 37 °C for 2 h, washed three times with fresh toluene, and dried. The thiol-terminated fiber was then immersed in 2 mM GMBS in PBS at room temperature for 1 h to introduce maleimide functionalities, washed three times with PBS, and immediately transferred to a 1 mg/mL CLA–BSA conjugate solution in PBS. Conjugate immobilization proceeded overnight at 4 °C with gentle agitation. Non-specific binding sites were blocked by incubation in 2 mg/mL BSA at 4 °C for 2 h. The functionalized bioprobes were washed three times with PBS and stored at 4 °C in PBS until use.

### 2.4. Preparation of Cy5.5-Labeled Anti-Clarithromycin Antibody

The anti-clarithromycin monoclonal antibody (clone CLA-4D3) was labeled with Cy5.5-SE. Cy5.5-SE stock solution was prepared according to the manufacturer’s instructions and stored at −80 °C. The antibody was buffer-exchanged into CBS (pH = 9.3) by repeated ultrafiltration (3000× *g*, 3 min, 4 °C) to ensure an amine-reactive environment. The dye-to-protein molar ratio was calculated according to the manufacturer’s protocol, and the appropriate volume of Cy5.5-SE working solution was added dropwise to the antibody under gentle stirring. The conjugation reaction was incubated at room temperature for 1 h while protected from light. Unreacted free dye was removed by repeated ultrafiltration in PBS (3000× *g*, 4 min, 4 °C) until the filtrate was colorless. The degree of labeling (DOL) and protein concentration were determined spectrophotometrically at 280 nm (protein absorbance) and 678 nm (Cy5.5 absorbance), respectively. The Cy5.5-labeled antibody was aliquoted and stored at −20 °C in the dark.

### 2.5. Indirect Enzyme-Linked Immunosorbent Assay

The affinity of the anti-clarithromycin antibody was characterized by indirect ELISA, and its specificity was characterized by indirect competitive ELISA. For affinity determination, microtiter plates were coated with CLA–BSA at three different concentrations (100 μL per well) and incubated overnight at 4 °C. After three washes with PBST, the wells were blocked with PBST containing 10% (*v*/*v*) calf serum at 37 °C for 2 h. Following additional PBST washes, serial dilutions of the anti-clarithromycin antibody were added (100 μL per well) and incubated at 37 °C for 45 min. For the competitive format, 50 μL of antibody and 50 μL of CLA standard (or structural analog) at varying concentrations were co-incubated in each well. After washing, HRP-labeled goat anti-mouse IgG (1:10,000 in PBST, 100 μL) was added and incubated at 37 °C for 30 min. The plates were washed again, and 100 μL of mixed substrate solution (TMB chromogen A and B, 1:1 *v*/*v*) was added. After 15 min of dark incubation at 37 °C, the reaction was terminated with 50 μL of 10% (*v*/*v*) H_2_SO_4_. Absorbance was measured at 450 nm using a microplate reader.

Affinity constant (*K*_a_) was calculated according to the method of Beatty et al. [32]:

(1) $$K_{a}=\frac{\left(n-1\right)}{2(n{Ab}^{\prime }-Ab)}$$

Here, *n* is the ratio of the two coating antigen concentrations (*n* > 1); *Ab*′ is the antibody concentration corresponding to half of the maximum absorbance (OD_max_/2) at one coating concentration; and *Ab* is that at the other coating concentration.

Cross-reactivity (*CR*) was calculated as:

(2) $$CR\;(\%)\;=\;\frac{{IC}_{50CLA}}{{IC}_{50others}}\;\times \;100$$

### 2.6. Biosensor Assay Protocol

The automated CLA detection workflow was executed on the evanescent wave biosensor platform according to the sequence summarized in Table S1. Before each assay, the fluidic system was primed with PBS to establish a stable fluorescence baseline, which was recorded as Ib. For indirect competitive detection, CLA standards or PBS were pre-mixed with the Cy5.5-labeled anti-clarithromycin antibody (5 μg/mL, optimized concentration) and incubated for a defined pre-reaction period to allow sufficient reaction between free CLA and the antibody. The pre-reacted mixture was then pumped into the flow cell housing the CLA–BSA-functionalized fiber bioprobe and incubated for 600 s, during which the remaining unbound antibody attached to the immobilized conjugate and was excited by the evanescent field. The fluorescence signal value after incubation was recorded as Is. After signal acquisition, the probe was regenerated by pumping 0.5% SDS (pH = 1.9) for 420 s to dissociate antigen–antibody complexes, followed by a 120 s PBS rinse to re-equilibrate the system. The entire cycle, from baseline to regeneration, was completed in approximately 20 min. Net fluorescence signals (Δ*Is*) were calculated as the difference between the post-incubation signal (*Is*) and the baseline signal (*Ib*):

(3) Δ*Is* = *Is* − *Ib*

### 2.7. Real-Sample Analysis

Tap water was collected from the Hebei Academy of Sciences (Shijiazhuang, China), and river water was collected from the Hutuo River in Shijiazhuang City, Hebei Province, China. Both samples were centrifuged (4000× *g*, 10 min) and filtered through 0.45 μm membrane filters to remove particulate matter, and then stored at 4 °C and analyzed within 30 days. For recovery experiments, CLA stock solution was spiked into the filtered water samples to achieve final concentrations of 10, 30, and 60 μg/L. Spiked samples were analyzed by the evanescent wave biosensor according to the protocol described in Section 2.6, and the results were cross-validated by indirect competitive ELISA performed in parallel.

## 3. Results

### 3.1. Antibody Characterization

**Figure 2:**
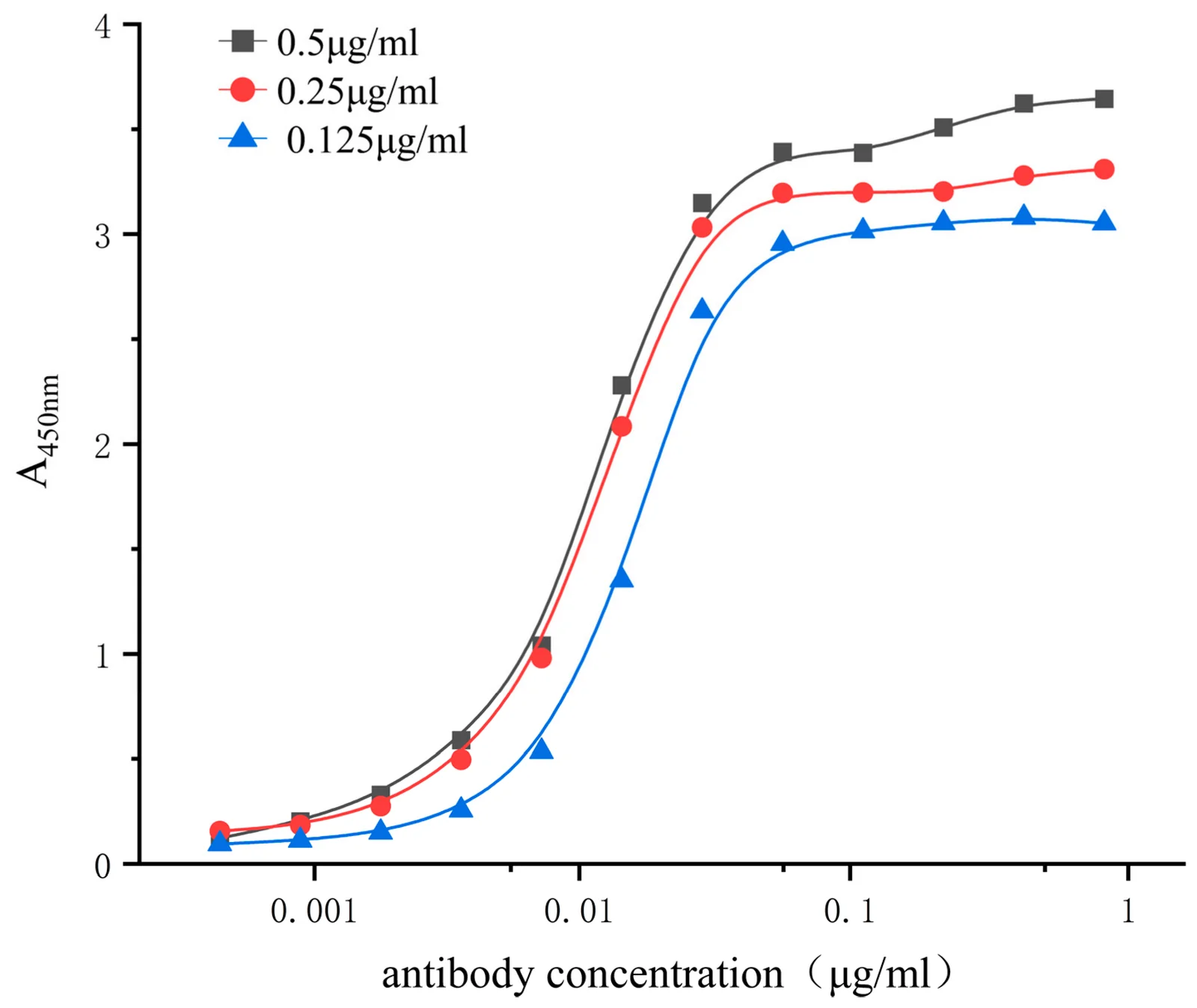
Affinity measurement results for the anti-clarithromycin antibody.

The affinity of the anti-clarithromycin monoclonal antibody (CLA-4D3) was determined by indirect ELISA using three coating antigen concentrations. As derived from Figure 2, the affinity constant *K*_a_ = 7.40 × 10^9^ L/mol. Based on the standard proposed by Harlow and Lane [33], this antibody possesses high-affinity properties.

The specificity of the antibody was evaluated against four structurally similar macrolides: telithromycin, azithromycin, erythromycin, and roxithromycin. As summarized in Table 1, the antibody exhibited negligible cross-reactivity with telithromycin (<0.08%), low cross-reactivity with azithromycin (3.89%), and moderate cross-reactivity with erythromycin (26.20%) and roxithromycin (31.66%). Overall, this antibody can effectively distinguish clarithromycin from its structural analogs, ensuring reliable detection specificity in complex environmental matrices where co-occurring macrolides may be present.

### 3.2. Cy5.5 Labeling Characterization

Successful conjugation of Cy5.5 to the anti-clarithromycin antibody was confirmed by UV–visible spectrophotometry (Table 2). The absorbance at 280 nm (protein) and 678 nm (Cy5.5 dye) was measured using a Hach DR6000 spectrophotometer, giving A_280_ = 0.470 and A_678_ = 0.634. These values yielded a DOL of 1.21 and a final labeled antibody concentration of 565.22 μg/mL. This value balances fluorescence brightness and retained immunoreactivity well.

### 3.3. Feasibility Analysis of CLA Detection Using the Evanescent Wave Biosensor

**Figure 3:**
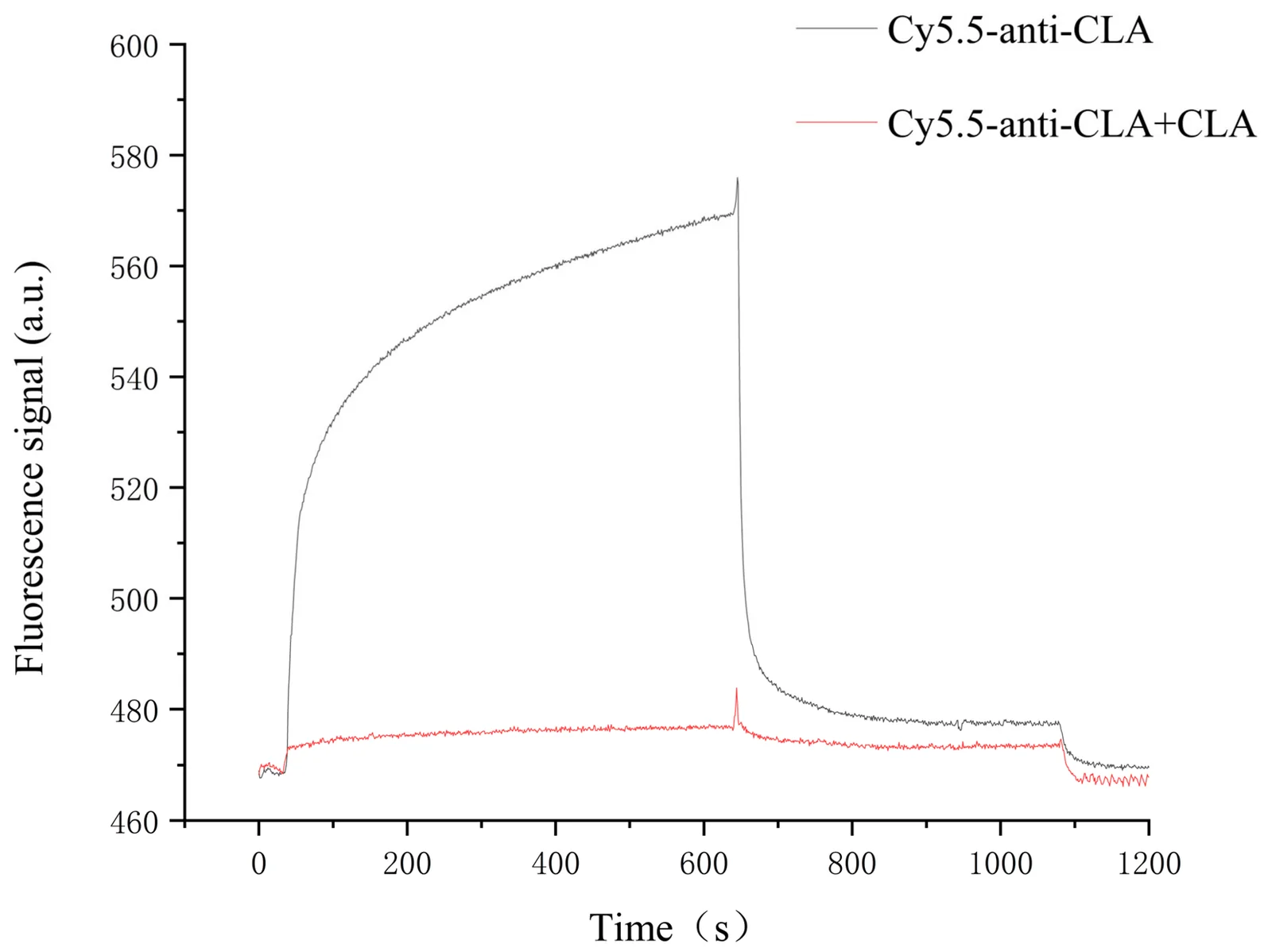
Fluorescence signal traces for 5 μg/mL Cy5.5-labeled anti-clarithromycin antibody alone and for the antibody mixed with 100 μg/L CLA.

The fundamental feasibility of the EW immunosensor for CLA detection was demonstrated by comparing the fluorescence responses of the Cy5.5-labeled antibody alone versus the antibody pre-incubated with excess free CLA (100 μg/L). As shown in Figure 3, introduction of the antibody-only sample produced a rapid fluorescence increase that plateaued within approximately 10 min, reflecting the specific binding of the labeled antibody to the immobilized CLA–BSA conjugate within the evanescent field. Subsequent regeneration with acidic SDS efficiently dissociated the bound antibody, returning the signal to baseline and confirming complete probe renewal. In contrast, the pre-mixed sample (antibody + 100 μg/L CLA) yielded a markedly attenuated signal, consistent with the competitive displacement of surface-bound binding by free CLA in solution. These observations validate the indirect competitive assay principle on the EW platform and confirm that both the surface immobilization chemistry and the fluorescent labeling preserve antigen–antibody recognition.

### 3.4. Optimization of Detection Conditions for CLA Using the Evanescent Wave Biosensor

Three critical parameters—antibody concentration, pre-reaction time, and immunoreaction time—were systematically optimized to maximize assay sensitivity and throughput. The antibody concentration was optimized using a sensitivity index (*ε*) defined as:

(4) $$\varepsilon =\frac{{\Delta \mathrm{Is}}_{b}-{\Delta \mathrm{Is}}_{s}}{{\Delta \mathrm{Is}}_{b}}$$

**Figure 4:**
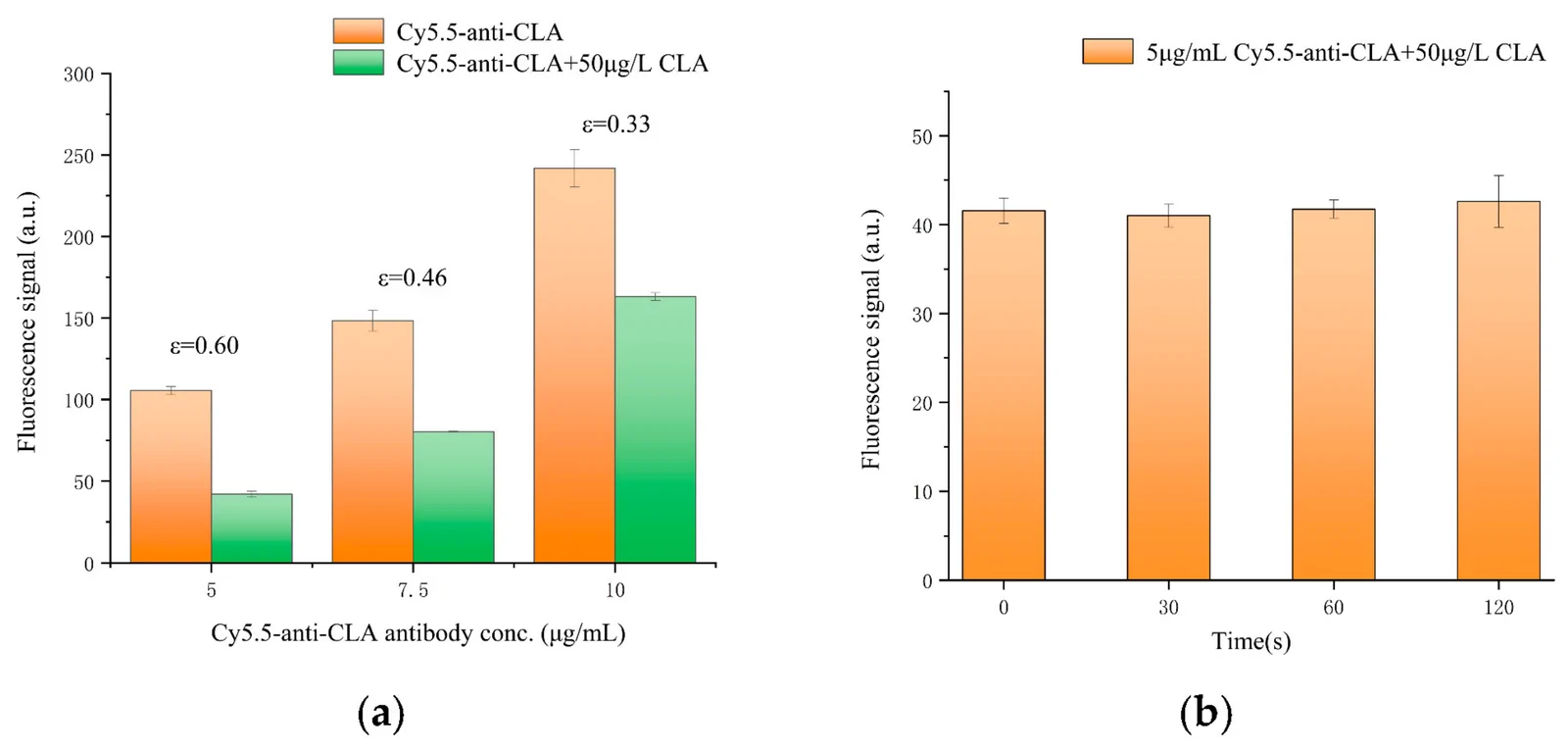
Optimization of detection conditions. (**a**) Cy5.5-labeled anti-clarithromycin antibody concentration: tested at 5, 7.5, and 10 μg/mL with a fixed clarithromycin (CLA) of 50.0 μg/L. (**b**) Pre-reaction time for the immunoassay: tested with 5 μg/mL labeled antibody and 50 μg/L.

Here, Δ*Is_b_* is the net fluorescence signal for a blank sample, and Δ*Is_s_* is that for a sample spiked with clarithromycin. In this study, the added clarithromycin concentration was 50.0 μg/L. As depicted in Figure 4a, *ε* decreased as the antibody concentration increased (5, 7.5, and 10 μg/mL), indicating that higher antibody concentrations reduce the sensitivity to clarithromycin. Although the absolute fluorescence signal (Δ*Is_b_*) increased with antibody concentration, the concomitant reduction in sensitivity favored the selection of 5 μg/mL as the working concentration, which provided the highest *ε* while maintaining adequate signal intensity. The pre-reaction time was evaluated at 0, 30, 60, and 120 s. As shown in Figure 4b, the fluorescence response remained largely invariant across the tested intervals, suggesting that the competitive binding equilibrium between free CLA and the antibody is established rapidly in homogeneous solution. To streamline the workflow without compromising performance, a 0 s pre-reaction was adopted for subsequent experiments. The immunoreaction time was evaluated from the fluorescence trace (Figure 3). The signal stabilized within 10 min. Therefore, a 10 min incubation time was chosen as the best compromise between assay speed and signal stability.

### 3.5. Establishment of the Standard Curve for CLA Detection

**Figure 5:**
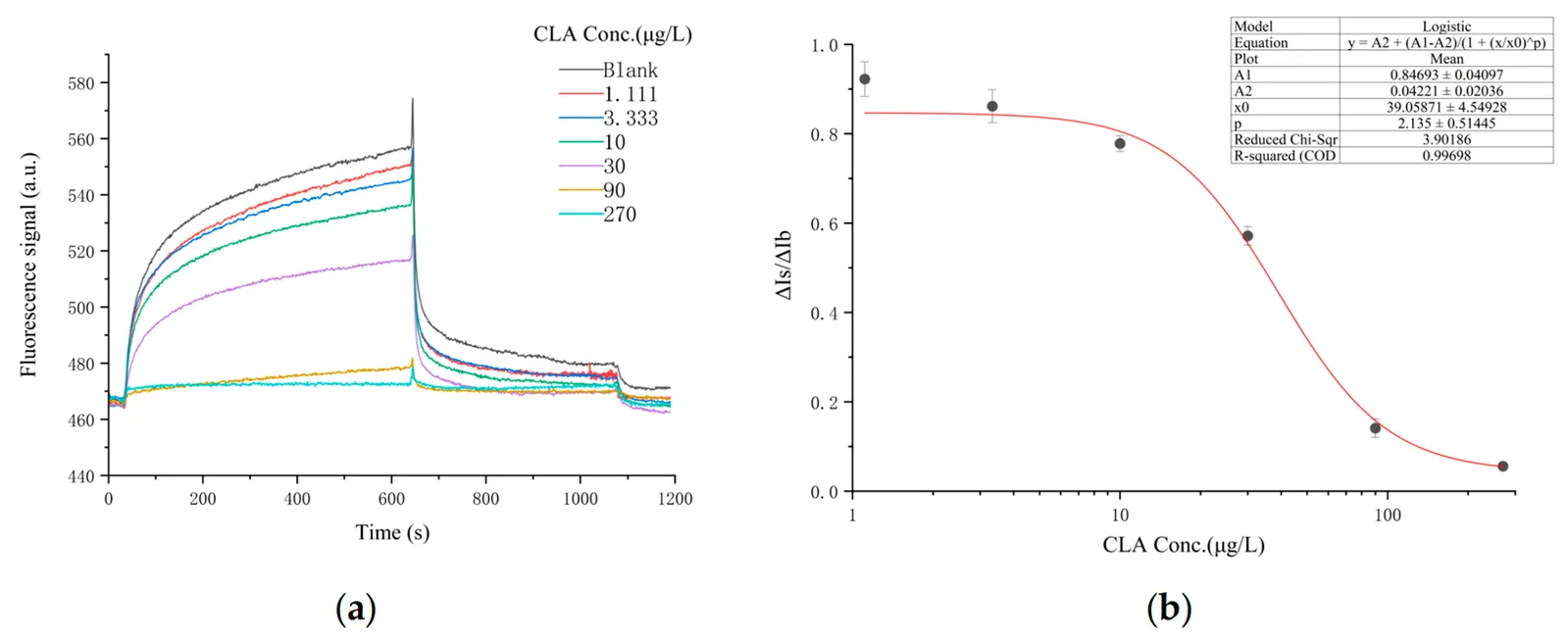
(**a**) Fluorescence signal traces for different clarithromycin concentrations using 5 μg/mL Cy5.5-labeled anti-clarithromycin antibody. (**b**) Standard calibration curve for clarithromycin obtained with 5 μg/mL Cy5.5-labeled anti-clarithromycin antibody.

Using the indirect competitive immunoassay principle, we can quantify clarithromycin. As depicted in Figure 5, increasing concentrations of the clarithromycin standard resulted in progressively stronger competition between free CLA and the immobilized CLA-BSA conjugate for binding to the Cy5.5-labeled antibody. Consequently, higher CLA concentrations led to greater inhibition of antibody–conjugate binding, accompanied by a corresponding decrease in the net fluorescence signal. Thus, an inverse relationship was established between the fluorescence signal and the CLA concentration.

The standard calibration curve for clarithromycin was fitted with a four-parameter logistic model using OriginPro 2025.

(5) $$y=A_{2}+\frac{(A_{1}-A_{2})\;}{\left[1+{\left(x/x_{0}\right)}^{p}\right]}$$

Here, *y* is the ratio of net fluorescence signals with and without clarithromycin; *x* is the clarithromycin concentration. *A*_1_ is the upper asymptote, and *A*_2_ is the lower asymptote. *x*_0_ is the inflection point—the concentration where the response changes most sharply. *p* is the slope factor. Under optimal conditions, the standard curve was established by incubating varying concentrations of clarithromycin with 5 μg/mL Cy5.5-labeled anti-clarithromycin antibody, followed by injection into the sample cell. The resulting fluorescence responses are presented in Figure 5a. The calibration curve (Figure 5b) yielded a working range of 7.50–77.45 μg/L, with the regression equation y = 0.847 + 0.805/[1 + (x/39.059)^2.135^] and R^2^ = 0.997. The limit of quantification (LOQ) and limit of detection (LOD) were determined to be 7.50 μg/L and 1.67 μg/L, respectively. Reproducibility was confirmed by a relative standard deviation (RSD) of less than 3.87%.

Collectively, these results indicate that the established calibration curve is well suited for the quantitative determination of clarithromycin at trace levels. Its detection limit meets the requirements for practical trace analysis.

### 3.6. Reusability and Stability of the Functionalized Fiber Bioprobe

**Figure 6:**
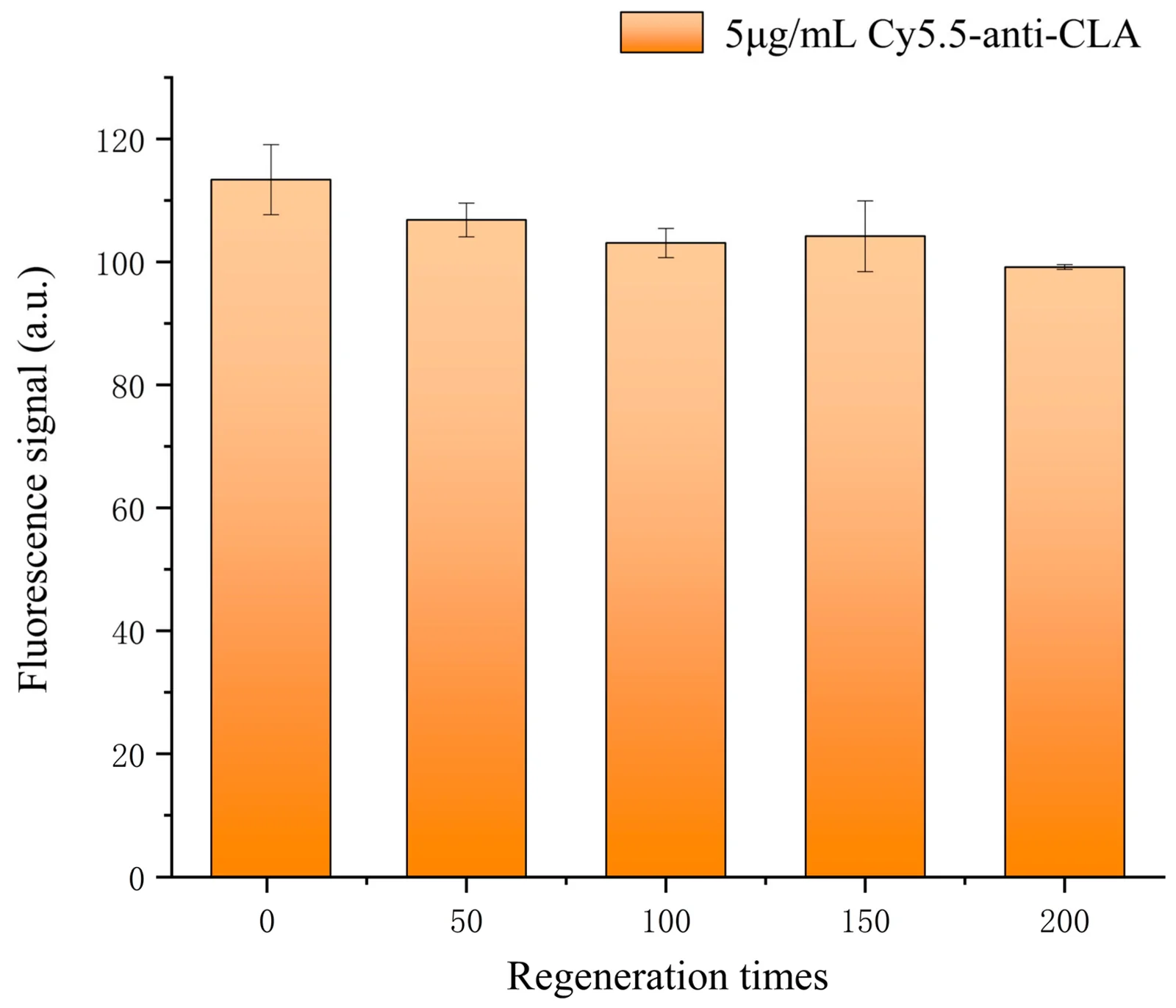
Fluorescence signal recorded with 5 μg/mL Cy5.5-labeled anti-clarithromycin antibody.

The reusability of the fiber bioprobe is a critical factor influencing the detection cost of the evanescent wave biosensor. Regeneration with SDS solution proved efficient, preserving the antigen–antibody binding activity and enabling multiple reuse cycles without appreciable loss of detection accuracy. As shown in Figure 6, the functionalized fiber bioprobe exhibited excellent stability, sustaining over 200 assay cycles with no significant signal attenuation and yielding a relative standard deviation (RSD) of 5.63%.

### 3.7. Detection of CLA in Water Samples

To validate the accuracy of the evanescent wave biosensor, tap water samples collected from the Hebei Academy of Sciences and river water samples from the Hutuo River in Shijiazhuang, Hebei Province, China, were analyzed. The samples were subjected to brief centrifugation and filtration prior to analysis. Clarithromycin standards were spiked into both tap water and river water samples at final concentrations of 10, 30, and 60 μg/L. As summarized in Table 3, the average recoveries ranged from 87.13% to 104.84%, with relative standard deviations (RSD) below 13.08% for all samples. The results were further compared with those obtained by enzyme-linked immunosorbent assay (ELISA) (Table S2), confirming that the proposed biosensor is reliable for the determination of clarithromycin in real water matrices, with good accuracy and precision and negligible matrix interference.

### 3.8. Performance Comparison with Reported Methods

We characterized the performance of our evanescent-wave fluorescence-based immunoassay for rapid clarithromycin testing. Its analytical figures of merit were compared with those of previously reported assays, as summarized in Table 4. The present immunosensor achieves a limit of detection (LOD) of 1.67 μg/L. Reusable optical-fiber probes make on-site measurement practical: the total assay time is under 20 min, sample pretreatment is minimal, and per-test costs are reduced. Conventional instrumental methods offer higher accuracy but require costly equipment, trained personnel, and lengthy sample preparation. ELISA achieves lower detection limits, though at the cost of substantially longer assay times. The electrophoresis–electrochemiluminescence method improves sensitivity through sweeping stacking injection, reaching an LOD of 30 nM. In comparison, our immunosensor requires no complex pretreatment and provides stable responses in environmental water matrices, making it promising for on-site screening.

## 4. Discussion

The evanescent wave fluorescence immunoassay presented here performed well in terms of precision, linearity (R^2^ = 0.997), and accuracy, with recoveries spanning 87.13–104.84%. Its instrumental limit of detection (LOD = 1.67 μg/L) and limit of quantification (LOQ = 7.50 μg/L) are, however, orders of magnitude above the ng/L concentrations usually encountered in surface water, and fall short of the sensitivity reached by the indirect competitive ELISA built on CLA-4D3. A likely reason is the comparatively high antibody concentration used during the detection step. Even so, the assay remains fit for purpose wherever analyte levels lie between μg/L and mg/L; no measurable interference was observed, and run-to-run reproducibility was high, both of which support its routine use. Coupling the sensor with a preconcentration step would be a natural next step, since this would push detection down to the ng/L range and allow long-term ecological effects to be gauged more reliably. Two further limitations deserve attention. First, matrix effects were examined only with conventional environmental water, so the anti-interference behaviour should be re-tested against real wastewater matrices. Second, refinements to the fluorescently labeled antibody would go some way towards making the device better suited to field monitoring.

## 5. Conclusions

In this work, we have developed a novel evanescent wave fluorescence immunosensor for the rapid, sensitive, and on-site detection of clarithromycin in environmental water samples. The sensor integrates a CLA–BSA conjugate-functionalized optical fiber bioprobe with a Cy5.5-labeled anti-clarithromycin monoclonal antibody (*K*_a_ = 7.40 × 10^9^ L/mol) as the signal reporter, operating on the indirect competitive immunoassay principle. Such elevated affinity is advantageous for evanescent wave biosensing because the evanescent field interrogates only molecular binding events occurring within the penetration depth (~100–200 nm); high-affinity interactions ensure rapid association, stable complex formation, and robust fluorescence signals. Under optimized conditions, the assay exhibited a detection limit of 1.67 μg/L and a linear working range of 7.50–77.45 μg/L, with excellent reproducibility (RSD < 3.87%). The functionalized fiber bioprobe demonstrated outstanding operational stability, sustaining over 200 regeneration cycles with negligible signal attenuation (RSD = 5.63%), which substantially reduces the per-assay cost and enhances the economic feasibility of the platform.

The combination of robust sensor stability, probe reusability, and cost-effectiveness renders this platform a promising candidate for rapid on-site detection of clarithromycin and environmental monitoring applications. Validation in tap water and river water matrices yielded spike recoveries ranging from 87.13% to 104.84% with RSD values below 13.08%, and the results were corroborated by ELISA, confirming negligible matrix interference and reliable accuracy. The entire automated detection workflow is completed within 20 min without manual intervention, offering a distinct advantage over conventional LC–MS/MS and ELISA methods in terms of portability, speed, and operational simplicity. Collectively, the high sensitivity, robust reusability, compact instrumentation, and user-friendly automation render this evanescent wave biosensor a promising analytical tool for on-site environmental surveillance of macrolide antibiotic residues, with substantial potential for extension to other emerging contaminants through appropriate antibody substitution.

## Figures and Tables

**Table 1:** Cross-reactivity of the anti-clarithromycin antibody against CLA and its analogs.

Antibiotic	IC_50_ (μg/L)	*CR* (%)
Clarithromycin	0.82	100
Telithromycin	>1000	<0.08
Azithromycin	21.10	3.89
Erythromycin	3.13	26.20
Roxithromycin	2.59	31.66

**Table 2:** Concentration and dye-to-protein ratio of the Cy5.5-labeled clarithromycin antibody.

Clarithromycin Antibody	Absorbance (Abs)		Degree of Labeling (DOL)	Concentration of Fluorescently Labeled Antibody (μg/mL)
CLA-4D3	280 nm	0.470	1.21	565.22
	678 nm	0.634		

**Table 3:** Spike recoveries of CLA in real water samples measured by the rapid evanescent wave fluorescence immunoassay.

Samples	Spiked (μg/L)	Detected (μg/L)	Recovery (%)	RSD% (n = 3)
tap water	10	10.3 ± 0.21	102.97	2.00
	30	26.74 ± 3.50	89.13	13.08
	60	61.27 ± 3.97	102.10	6.47
river water	10	10.48 ± 0.16	104.84	1.50
	30	26.14 ± 1.54	87.13	5.88
	60	61.53 ± 4.48	102.56	7.28

**Table 4:** Comparison of the proposed immunosensor with other reported methods for CLA detection.

Method	LOD	Assay Time	Sample Pretreatment	Testing Cost	References
Evanescent wave immunosensor	1.67 μg/L	<20 min	Centrifuge, filter	low	This work
HPLC	5–6 µg/L	~7 min	Centrifugation, filtration	Medium	[34]
LC-MS	30 µg/L	30 min	Protein precipitation, liquid–liquid extraction	High	[15]
ELISA	0.02–0.03 μg/L	~15 h	Dilution	Medium	[17]
Electrophoresis–electrochemiluminescence	30 nM	~40 min	vortex oscillation	Medium-low	[35]

## Data Availability

All the data generated during this study are included in the manuscript. Additional data related to this article may be requested from the corresponding authors.

## Supplementary Materials

The following supporting information can be downloaded at: https://www.mdpi.com/article/10.3390/bios16100588/s1. Figure S1: Indirect competitive binding curves of different macrolide antibiotics; Table S1: CLA detection procedure using the evanescent wave biosensor; Table S2: Intra- and inter-batch recoveries and coefficients of variation for clarithromycin in spiked water samples using the ELISA method (n = 6).

## Abbreviations

The following abbreviations are used in this manuscript:

CLA	Clarithromycin
BSA	Bovine Serum Albumin
LOD	Limit of Detection
LOQ	Limit of Quantitation
ELISA	Enzyme-Linked Immunosorbent Assay

## References

[1] Davidson R.J. (2019). In vitro activity and pharmacodynamic/pharmacokinetic parameters of clarithromycin and azithromycin: Why they matter in the treatment of respiratory tract infections. Infect. Drug Resist..

[2] Akinosoglou K., Leventogiannis K., Tasouli E., Kakavoulis N., Niotis G., Doulou S., Skorda L., Iliopoulou K., Papailiou A., Katsaounou P. (2025). Clarithromycin for improved clinical outcomes in community-acquired pneumonia: A subgroup analysis of the ACCESS trial. Int. J. Antimicrob. Agents.

[3] Parish L.C. (1993). Clarithromycin in the treatment of skin and skin structure infections: Two multicenter clinical studies. Clarithromycin Study Group. Int. J. Dermatol..

[4] Guay D.R., Gustavson L.E., Devcich K.J., Zhang J., Cao G., Olson C.A. (2001). Pharmacokinetics and tolerability of extended-release clarithromycin. Clin. Ther..

[5] Gotfried M.H. (2003). Clarithromycin (Biaxin) extended-release tablet: A therapeutic review. Expert Rev. Anti-Infect. Ther..

[6] Parsad D., Pandhi R., Dogra S. (2003). A guide to selection and appropriate use of macrolides in skin infections. Am. J. Clin. Dermatol..

[7] Miroshnyk I., Mirza S., Zorky P.M., Heinämäki J., Yli-Kauhaluoma J., Yliruusi J. (2008). A new insight into solid-state conformation of macrolide antibiotics. Bioorg. Med. Chem..

[8] Della-Negra O., Bastos M.C., Bru-Adan V., Santa-Catalina G., Ait-Mouheb N., Chiron S., Patureau D. (2025). Temporal dynamics of the soil resistome and microbiome irrigated with treated wastewater containing clarithromycin. Environ. Res..

[9] Haenni M., Dagot C., Chesneau O., Bibbal D., Labanowski J., Vialette M., Bouchard D., Martin-Laurent F., Calsat L., Nazaret S. (2022). Environmental contamination in a high-income country (France) by antibiotics, antibiotic-resistant bacteria, and antibiotic resistance genes: Status and possible causes. Environ. Int..

[10] Ashbolt N.J., Amézquita A., Backhaus T., Borriello P., Brandt K.K., Collignon P., Coors A., Finley R., Gaze W.H., Heberer T. (2013). Human Health Risk Assessment (HHRA) for environmental development and transfer of antibiotic resistance. Environ. Health Perspect..

[11] Peris-Vicente J., Peris-García E., Albiol-Chiva J., Durgbanshi A., Ochoa-Aranda E., Carda-Broch S., Bose D., Esteve-Romero J. (2022). Liquid chromatography, a valuable tool in the determination of antibiotics in biological, food and environmental samples. Microchem. J..

[12] Yan Z., Huang X., Xie Y., Song M., Zhu K., Ding S. (2019). Macrolides induce severe cardiotoxicity and developmental toxicity in zebrafish embryos. Sci. Total Environ..

[13] Ibekwe A.M., Gonzalez-Rubio A., Suarez D.L. (2018). Impact of treated wastewater for irrigation on soil microbial communities. Sci. Total Environ..

[14] Tian B., Zhang M., Zhu C., Yang R., Yin G., Hu S., Chen Y., Zhao N. (2025). Contrastive cognition into the occurrence, source identification and risk assessment of antibiotics in various drinking water sources. Environ. Pollut..

[15] Zouari M., Cetinkaya A., Ozkan S.A. (2025). Comparative analysis of LC-MS/MS and electrochemical immunosensor for clarithromycin detection in plasma. Microchim. Acta.

[16] Chen J., Mei G., Zhang X., Huang D., He P., Xu D. (2024). Dispersive Solid-Phase Extraction and Ultra-Performance Liquid Chromatography-Tandem Mass Spectrometry-A Rapid and Accurate Method for Detecting 10 Macrolide Residues in Aquatic Products. Foods.

[17] Galvidis I., Lapa G., Burkin M. (2015). Group determination of 14-membered macrolide antibiotics and azithromycin using antibodies against common epitopes. Anal. Biochem..

[18] Manoharan Nair Sudha Kumari S., Thankappan Suryabai X. (2024). Sensing the Future-Frontiers in Biosensors: Exploring Classifications, Principles, and Recent Advances. ACS Omega.

[19] Liu Z., Yang W., Lin H., Zhang M., Sun C. (2025). Recent Advances of Fluorescent Aptasensors for the Detection of Antibiotics in Food. Biosensors.

[20] Lam S.Y., Lau H.L., Kwok C.K. (2022). Capture-SELEX: Selection Strategy, Aptamer Identification, and Biosensing Application. Biosensors.

[21] Bian J., Cai S., Chen X., Wang X., Zhang Y., Zhou N. (2026). A smartphone-integrated portable fluorescent biosensor for on-site detection of antibiotics. Anal. Chim. Acta.

[22] Wang M., Liu S., Lu L., Liu J., Long F., Li C. (2025). Field-deployable automated evanescent wave fluorescence optofluidic biochip for ultrasensitive detection of clenbuterol residues in complex animal urines. Chem. Eng. J..

[23] Long F., He M., Shi H.C., Zhu A.N. (2008). Development of evanescent wave all-fiber immunosensor for environmental water analysis. Biosens. Bioelectron..

[24] Chen C., Singh R., Huo S., Song Y., Wang K., Chiavaioli F., Hou X. (2026). Evanescent wave-based optical biosensors for innovations, medical application and future perspectives. J. Adv. Res..

[25] Nedoma J., Krizan D., Stipal J., Pereira L., Bekmurzayeva A., Tosi D., Costa M.B., Leal-Junior A., Martinek R., Marques C. (2026). Decade of advancements in light-matter interaction-based optical fiber biosensing: Innovations, challenges, and future directions. Adv. Photonics.

[26] Song D., Xu W., Han X., Wang H., Zhuo Y., Liu J., Zhu A., Long F. (2023). CRISPR/Cas12a-powered evanescent wave fluorescence nanobiosensing platform for nucleic acid amplification-free detection of Staphylococcus aureus with multiple signal enhancements. Biosens. Bioelectron..

[27] Han X., Song D., Xu W., Lu L., Zhu A., Long F. (2024). CRISPR/Cas12a powered air-displacement enhanced evanescent wave fluorescence fiber-embedded microfluidic biochip for nucleic acid amplification-free detection of *Escherichia coli* O157:H7. J. Hazard. Mater..

[28] Yi Z., Zhou Y., Ren Y., Hu W., Long F., Zhu A. (2022). A novel sensitive DNAzyme-based optical fiber evanescent wave biosensor for rapid detection of Pb^2+^ in human serum. Analyst.

[29] Zhou Y., Wang H., Song D., Li Z., Han S., Long F., Zhu A. (2021). Simple, rapid, and sensitive on-site detection of Hg(2+) in water samples through combining portable evanescent wave optofluidic biosensor and fluorescence resonance energy transfer principle. Anal. Chim. Acta.

[30] Li Y., Xu W., Liu J., Zhang E., Li H., Zhang Y., Zhang J., Li C., Zhang X. (2023). Rapid and Sensitive On-Site Detection of Fipronil in Foods Using Evanescent Wave Fluorescent Immunosensor. Chemosensors.

[31] Zhuo Y., Xu W., Cheng Y., Song D., Han X., Long F. (2022). Rapid detection of norfloxacin by evanescent-wave optical-fiber biosensor. China Environ. Sci..

[32] Beatty J.D., Beatty B.G., Vlahos W.G. (1987). Measurement of monoclonal antibody affinity by non-competitive enzyme immunoassay. J. Immunol. Methods.

[33] Harlow E., Lane D. (1988). Antibodies: A Laboratory Manual.

[34] Duong T.T.A., Vuong T.X., Ta T.T., Nguyen T.T.L., Ngo T.M.V. (2025). Development of an HPLC method for the simultaneous determination of azithromycin, clarithromycin, and erythromycin in wastewater. RSC Adv..

[35] Peng X., Wang Z., Li J., Le G., Shi Y. (2008). Electrochemiluminescence Detection of Clarithromycin in Biological Fluids after Capillary Electrophoresis Separation. Anal. Lett..

