# Supplementary material for: Rapid On-Site Detection of Clarithromycin in Water Using an Evanescent Wave Fluorescence Immunosensor
Source: Biosensors (Basel). 2026 Oct 20;16(10):588. doi: 10.3390/bios16100588 (PMC13702604; doi:10.3390/bios16100588)
Supplement: Supplementary file 1 [file biosensors-16-00588-s001.zip › biosensors-4600250-supplementary.pdf]

Supplementary Information

Evaluation of antibody specificity by indirect competitive ELISA

Indirect competitive binding assays were carried out for clarithromycin, telithromycin, azithromycin, erythromycin, and roxithromycin. The corresponding binding curves are shown in Figure S1. The IC_{50} values of clarithromycin, telithromycin, azithromycin, erythromycin and roxithromycin were 0.82, >1000, 21.10, 3.13 and 2.59 μg/L, respectively.

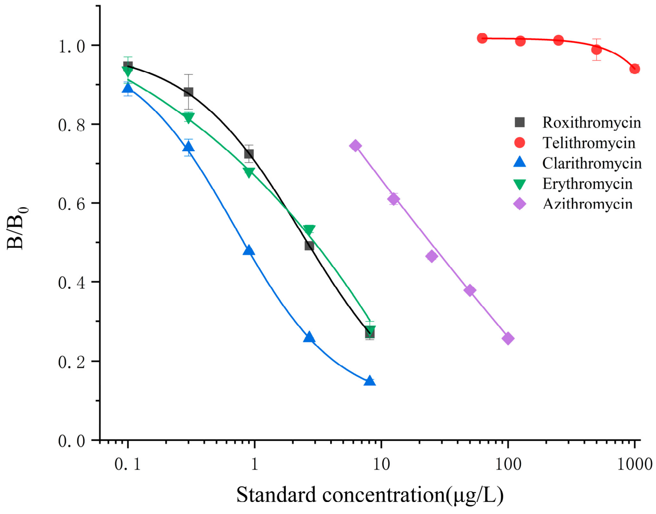

Figure S1 Indirect competitive binding curves of different macrolide antibiotics

The detection process of the evanescent wave biosensor

An automated rapid detection procedure for clarithromycin was established, which completed the steps listed in the table below within approximately 20 min without manual intervention. A mini-computer was programmed to coordinate the multi-way valve and the peristaltic pump throughout the process.

Table S1 CLA detection procedure using the evanescent wave biosensor

Step	Channel	Solution	Time (s)	Function
Baseline	1	Buffer	40	Stabilize the baseline

incubate	6	Pre-reacted sample solution	600	Allow immunoreaction for full antigen-antibody binding
Regeneration	4	Regeneration solution	420	Dissociate antigen-antibody complexes and elute antibodies
rinsing	1	Buffer	120	Rinse the tubing and re-equilibrate the system

Recovery experiment by spiking in enzyme-linked immunosorbent assay

Clarithromycin standards were spiked into tap water and river water samples at final concentrations of 1 μg/L, 5 μg/L, and 10 μg/L, respectively. The intra- and inter-assay recoveries for tap water samples ranged from 86.77% to 115.07%, with coefficients of variation (CVs) below 7.68%. For river water samples, the recoveries ranged from 80.90% to 117.96%, with CVs below 10.53%. These findings demonstrate that the proposed method has high accuracy and precision.

Table S2 Intra- and inter-batch recoveries and coefficients of variation for clarithromycin in spiked water samples using the ELISA method (n = 6)

Sample	Spiked concentration (μg/L)	Recovery (%)	Intra-batch CV (%)	Inter-batch CV (%)
Tap water	1	92.67±6.38	6.88	7.67
		101.71±7.81	7.68	
		99.18±6.12	6.17	
	5	115.07±7.30	6.35	5.58
		110.70±4.00	3.61	
		111.76±7.24	6.47	
	10	86.77±2.78	3.20	6.80
		94.89±4.90	5.16	
		98.83±3.87	3.92	
River water	1	87.70±3.36	3.84	7.55
		88.04±9.27	10.53	
		80.90±2.34	2.89	
	5	109.04±2.33	2.13	6.33
		111.92±7.91	7.07	
		104.23±7.48	7.18	
	10	109.97±4.59	4.18	7.37
		117.96±9.09	7.70	
		110.71±9.21	8.32	
